# Supplementary material for: MetaQTL: a package of new computational methods for the meta-analysis of QTL mapping experiments
Source: BMC Bioinformatics. 2007 Feb 8;8:49. doi: 10.1186/1471-2105-8-49 (PMC1808479; doi:10.1186/1471-2105-8-49)
Supplement: Additional File 7 — MetaQTL Package : jar file and tutorial. This Zip archive contains both the MetaQTL JAR file and the files of the tutorial. [file 1471-2105-8-49-S7.zip › org.inra.metaqtl/doc/org/metaqtl/graph/AnchoredElement.html]

AnchoredElement


|  |  |  |
| --- | --- | --- |
| **Overview** \| **Package** \| **Class** \| **Use** \| **Tree** \| **Deprecated** \| **Index** \| **Help** | |  |
| PREV CLASS   **NEXT CLASS** | **FRAMES**    **NO FRAMES**     **All Classes** |  |
| SUMMARY: NESTED \| FIELD \| CONSTR \| METHOD | DETAIL: FIELD \| CONSTR \| METHOD |  |


---


## org.metaqtl.graph Class AnchoredElement

```
java.lang.Object
  org.metaqtl.graph.AnchoredElement
```

**Direct Known Subclasses:**: Layer

---

``` public abstract class AnchoredElement extends java.lang.Object ```

---

| **Field Summary** | |
| --- | --- |
| `protected  double` | `x` |
| `protected  double` | `y` |


**Constructor Summary**

`AnchoredElement()`


| **Method Summary** | |
| --- | --- |
| `double` | `getX()` |
| `double` | `getY()` |
| `void` | `setX(double x)` |
| `void` | `setY(double y)` |

**Methods inherited from class java.lang.Object**

`clone, equals, finalize, getClass, hashCode, notify, notifyAll, toString, wait, wait, wait`

**Field Detail**

### x

```
protected double x
```

---


### y

```
protected double y
```


**Constructor Detail**

### AnchoredElement

```
public AnchoredElement()
```


**Method Detail**

### getX

```
public double getX()
```

:   **Returns:**: Returns the x.

---


### getY

```
public double getY()
```

:   **Returns:**: Returns the y.

---


### setX

```
public void setX(double x)
```

:   **Parameters:**: `x` - The x to set.

---


### setY

```
public void setY(double y)
```

:   **Parameters:**: `y` - The y to set.


---


|  |  |  |
| --- | --- | --- |
| **Overview** \| **Package** \| **Class** \| **Use** \| **Tree** \| **Deprecated** \| **Index** \| **Help** | |  |
| PREV CLASS   **NEXT CLASS** | **FRAMES**    **NO FRAMES**     **All Classes** |  |
| SUMMARY: NESTED \| FIELD \| CONSTR \| METHOD | DETAIL: FIELD \| CONSTR \| METHOD |  |


---
